# Use of Spatial Information to Predict Multidrug Resistance in Tuberculosis Patients, Peru

**DOI:** 10.3201/eid1805.111467

**Published:** 2012-05

**Authors:** Hsien-Ho Lin, Sonya S. Shin, Carmen Contreras, Luis Asencios, Christopher J. Paciorek, Ted Cohen

**Affiliations:** Brigham and Women’s Hospital, Boston, Massachusetts, USA (H.H. Lin, S.S. Shin, T. Cohen);; Mennonite Christian Hospital, Hualien, Taiwan (H.H. Lin);; National Taiwan University, Taipei, Taiwan (H.H. Lin);; Partners In Health, Boston (S.S. Shin);; Socios En Salud Sucursal Peru, Lima, Peru (C. Contreras);; Instituto Nacional de Salud, Lima (L. Asencios);; Harvard School of Public Health, Boston (C.J. Paciorek, T. Cohen);; University of California, Berkeley, California, USA (C.J. Paciorek)

**Keywords:** Tuberculosis and other mycobacteria, TB, tuberculosis, antimicrobial resistance, multidrug resistance, MDR, multidrug-resistant, geographic information systems, GIS, drug susceptibility testing, DST, Peru, Lima

## Abstract

To determine whether spatiotemporal information could help predict multidrug resistance at the time of tuberculosis diagnosis, we investigated tuberculosis patients who underwent drug susceptibility testing in Lima, Peru, during 2005–2007. We found that crude representation of spatial location at the level of the health center improved prediction of multidrug resistance.

## Abstract 2

Knowing whether a patient has multidrug-resistant tuberculosis is crucial for prescribing the best treatment. The challenge is choosing the most effective drug with the fewest side effects while saving the “big guns” for the most resistant infections. The best way to find out whether a patient has this type of infection is to conduct drug-susceptibility testing. Unfortunately, this testing requires laboratory capabilities that are in short supply, so often only patients at high risk are tested. But who is at high risk? A recent study found an association between patients’ locations (health center at which they were seen) and likelihood of multidrug-resistant infection. Added to other known risk factors (young age, previous TB treatment, or contact with someone with similar infection), this information can further pinpoint who should be tested, which will ultimately lead to faster diagnoses, better treatments and less spread of multidrug-resistant TB.

In many locations where risk for tuberculosis (TB) is high, access to drug-susceptibility testing (DST) is limited. The detection of drug resistance in these instances usually requires the use of culture-based DST, but laboratory capacity in these areas is in short supply. As a result, DST is rationed, with patients at highest risk for drug resistance receiving priority. New rapid tests for resistance that circumvent some constraints are being implemented, and universal DST might eventually be available (*1*); however, most clinicians in high-risk areas will not have access to these tools for at least several years. Accordingly, improved prediction of risk for multidrug-resistant (MDR) TB, defined as resistance to at least isoniazid and rifampin, might reduce delay to appropriate diagnosis, improve treatment outcomes, and decrease the risk for MDR TB transmission.

Demographic and clinical characteristics that have been associated with increased risk for MDR TB among patients with incident TB are young age, previous TB treatment, and known contact with MDR TB (*2**,**3*). In the context of limited access to DST, these risk factors are often incorporated into diagnostic algorithms to help justify use of DST. We hypothesized that information about the location and time at which cases were detected might also improve prediction of MDR TB (*3**–**5*). We analyzed programmatic data collected in Lima, Peru, about TB patients who were receiving DST to assess whether predictive models that include information about time and location could improve prediction of risk for MDR TB.

## The Study

**Figure 1:**
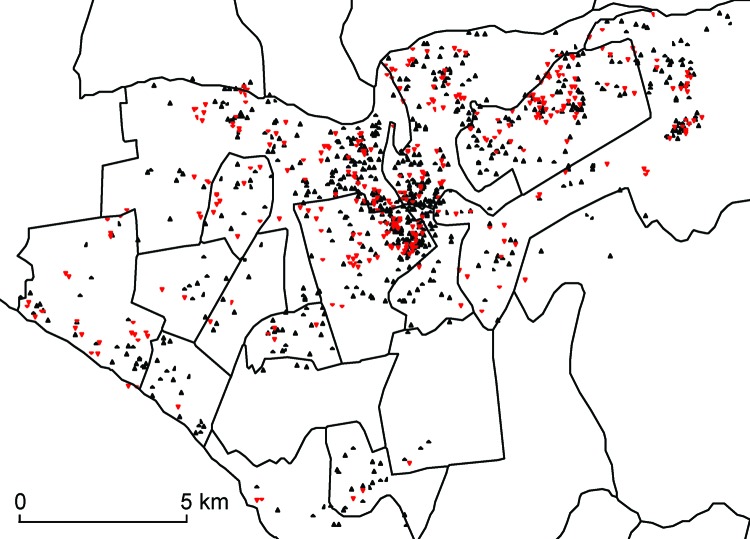
Spatial distribution of drug-sensitive (black triangles) and drug-resistant (red triangles) tuberculosis among patients who received drug susceptibility testing, Lima Ciudad and Lima Este, Peru, 2005–2007. A small random error was added to the spatial coordinates for each patient to protect confidentiality.

We selected our study population from among all 11,711 patients with reported cases of TB in 2 of Lima’s 4 health districts, Lima Ciudad and contiguous catchment areas of Lima Este, during January 1, 2005–December 31, 2007. Demographic and clinical information about these patients was collected from routine TB program data. The home addresses of the patients were geocoded by using high-resolution maps created in Google Earth (Google Inc., Mountain View, CA, USA). In Peru, only a subset of TB patients determined to be at high risk for MDR TB receive sputum culture and DST; consistent with local guidelines, these patients are those who had previous TB treatment, known household contact with MDR TB patients, or lack of response to first-line TB treatment (*6*). We limited our analyses to patients who underwent DST and who had a definitive positive or negative result (n = 1,116); 346 of these patients had MDR TB (Figure 1). Additional study details are provided in Lin et al. (*7*).

To identify risk factors for MDR TB, we constructed a logistic regression model that included age, sex, sputum smear test result, previous TB treatment, known household contact with MDR TB patients, and HIV infection status as potential predictors. Univariable analyses showed that age at diagnosis, history of TB treatment, and sputum smear–negative disease were significantly associated with risk for MDR TB (Table). In the multivariable adjusted analysis, age at diagnosis, history of TB treatment, sputum smear–negative disease, and HIV-positive status were found to be independent predictors of MDR TB (Table).

**Table:** Risk for MDR TB among TB patients who received DST, by demographic and clinical characteristics, Lima Ciudad and Lima Este, Peru, 2005–2007 *

Characteristic	Univariate OR† (95% CI)	p value	Multivariate OR† (95% CI)‡	p value
Age, per 10-y increase	0.89 (0.81–0.99)	0.034	0.90 (0.81–1.00)	0.046
Male sex	1.09 (0.81–1.46)	0.58	1.05 (0.78–1.43)	0.74
Negative sputum smear test results	2.05 (1.37–3.08)	<0.001	2.11 (1.40–3.19)	<0.001
HIV infection	0.59 (0.31–1.13)	0.11	0.52 (0.27–1.00)	0.049
History of TB treatment	2.38 (1.78–3.18)	<0.001	2.41 (1.80–3.23)	<0.001
Known household contact with persons with MDR TB	1.18 (0.89–1.57)	0.25	1.08 (0.81–1.44)	0.59

*MDR, multidrug resistant; TB, tuberculosis; DST, drug-susceptibility testing; OR, odds ratio. †ORs represent risk for MDR TB conditional on receiving DST. For example, even if household contact with persons with MDR TB is marginally associated with increased risk for MDR TB, the observed association would likely be attenuated when the analysis is restricted to those who received DST because household contact with persons with MDR TB is an accepted criterion for ordering DST. ‡Adjusted for all other variables listed.

To determine whether spatiotemporal information improved prediction of MDR TB, we further constructed 3 spatial regression models: 1) a health center model that combined demographic and clinical factors with health center information, modeled as random intercepts (*8*); 2) a spatial model that combined demographic and clinical factors with individual-level spatial information (i.e., patient residence), modeled as a smooth term using thin-plate regression splines (*9*); and 3) a spatiotemporal model that combined demographic and clinical factors with individual-level spatiotemporal information (i.e., patient residence and date of TB diagnosis), modeled as a smooth term using thin-plate regression splines (*10*). We compared model performance of the 3 spatial models against a nonspatial model, which comprised only demographic and clinical factors.

To evaluate the accuracy of the models, we held out the last 50% of cases according to diagnosis date and used the first 50% of cases to fit the models. We then made predictions on the held-out cases by using the fitted models; receiver operating characteristic (ROC) analysis was used to estimate the area under the curve (AUC) for the held-out cases under each of the 4 models. We also computed the logistic regression likelihood (Bernoulli density) of the held-out data; the model with the largest logistic regression likelihood was judged to be most accurate (*11*).

**Figure 2:**
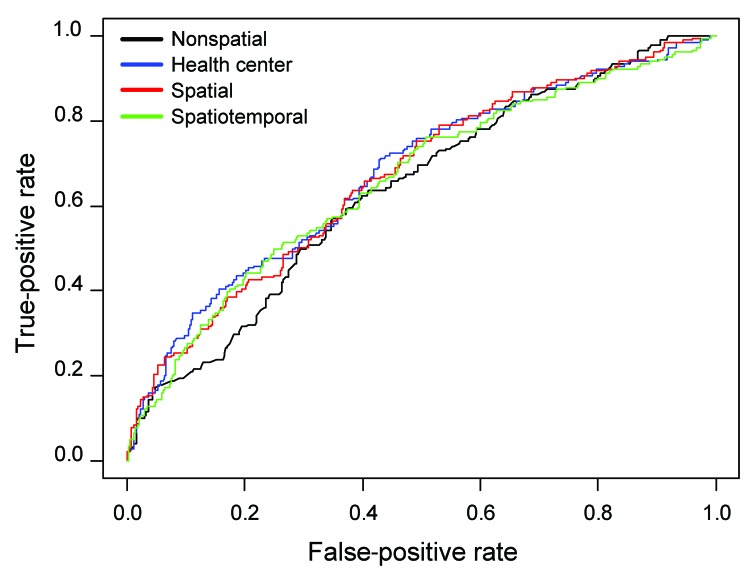
Receiver operating characteristic curves for the 4 prediction models for multidrug-resistant tuberculosis among patients who received drug susceptibility testing, Lima Ciudad and Lima Este, Peru, 2005–2007.

The ROC analysis suggested that the addition of spatial information improved the performance of the nonspatial model (Figure 2). The AUC for the nonspatial model was 0.64 (95% CI 0.59–0.69, compared with 0.67 (95% CI 0.63–0.72) for the health center model (p = 0.02 for comparison with the nonspatial model); 0.67 (95% CI 0.62–0.72) for the spatial model (p = 0.06 for comparison with the nonspatial model); and 0.66 (95% CI 0.61–0.71) for the spatiotemporal model (p = 0.36 for comparison with the nonspatial model). The logarithm of logistic regression likelihood for the spatial model (−328.1) and the health center model (−327.0) were greater than that of the nonspatial model (−335.1), which suggests that the use of spatial information improved predictive power.

## Conclusions

In locations where capacity is not available to provide DST for all patients with incident TB, improved methods to predict MDR TB at the time of diagnosis would be valuable. We found that information about location (represented as either the health center of diagnosis or the patient’s residence location) improved prediction of MDR TB among those who received DST. Whereas the improvement in the models was either statistically significant (comparing health center and nonspatial models) or trending toward significance (comparing spatial and nonspatial models), the absolute differences in the AUCs from spatial and nonspatial models were modest. Despite the minor improvements, spatial and temporal information may be useful for targeting testing when access is limited. From a practical standpoint, these results suggest that adopting more lenient criteria for ordering DST for TB patients at individual health centers where risk for MDR TB is highest may be a rational approach while resources are limited.

Models with simple representations of space (i.e., identification of location only at the level of the health center) outperformed models that captured spatial risk in finer spatial resolution. This finding is consistent with an earlier analysis in which we found relative aggregation of new MDR TB at a spatial scale of 4–7 km (*7*). Together, these results suggest dispersed spatial risk for resistance in the study area, which indicates that, from a public health perspective, policies prioritizing the use of DST for patients originating from large administrative areas may be helpful.

Because we could include only patients who received DST, we can make inference only among this subgroup of patients. However, if use of DST were randomized throughout the study area (as earlier analysis suggests [*7*]), inference from this subgroup should be generalizable to all patients with incident TB. Use of historical data for spatial prediction relies on the assumption that the spatial patterns remain constant or change in a predictable manner. Temporal changes in spatial distribution of MDR TB would have reduced the predictive ability of the models, yet we found that spatial information improved our predictions. Further research is warranted to test this approach in settings where the spatial pattern of TB differs from that of Lima, preferably by using datasets in which DST has been conducted for all TB patients to prevent potential sampling bias.
